# QUADRICEPS MUSCLE THICKNESS MEASURED BY POINT-OF-CARE ULTRASOUND AND HOSPITAL LENGTH OF STAY

**DOI:** 10.1093/geroni/igae098.2612

**Published:** 2024-12-31

**Authors:** Uyanga Ganbat, Altan-Ochir Byambaa, Graydon Meneilly

**Affiliations:** University of British Columbia, Vancouver, British Columbia, Canada; University of British Columbia, Vancouver, British Columbia, Canada; University of British Columbia, Vancouver, British Columbia, Canada

## Abstract

**Background:**

Accurate prediction of hospital length of stay (LOS) and readmission rates could help improve effective healthcare resource allocation. Recent evidence suggests point-of-care ultrasound for muscle assessment, specifically muscle thickness, as a promising tool in this regard. This study explores the hypothesis that ultrasound measurements of quadriceps muscle thickness (MT) and echointensity (EI) can serve as predictors for these crucial patient outcomes.

**Methods:**

The study measured quadriceps MT and EI using point-of-care ultrasound for patients in a supine position in the emergency department of Vancouver General Hospital. Predictor variables included age, sex, muscle thickness, and echo intensity. Outcome variables were hospital LOS, readmission rate, and discharge destination. Follow-up was conducted after one month to assess hospital readmissions and mortality.

**Results:**

A total of 120 participants were included (average age 76.9 ± 7.5, with 64 women and 56 men). Mean LOS was 27.4 ± 31.4 days, and mean MT was 20 ± 6 mm. Sex-based differences in MT were statistically significant (P = 0.032). MT correlated significantly with LOS (Standardized β = -0.152 ± 0.016, R² = 0.290, P = 0.001). In the multivariate regression model, MT remained a significant predictor (Standardized β = -0.152 ± 0.563, P = 0.008).

**Conclusion:**

Muscle thickness is a significant predictor of hospital stay duration. The findings of this study indicate the potential of point-of-care ultrasound in measuring skeletal muscle as an effective predictor of discharge outcomes.

